# Influencing factors of multiple adverse outcomes among schizophrenia patients using count regression models: a cross-sectional study

**DOI:** 10.1186/s12888-022-04070-3

**Published:** 2022-07-15

**Authors:** Lichang Chen, Wenyan Tan, Xiao Lin, Haicheng Lin, Junyan Xi, Yuqin Zhang, Fujun Jia, Yuantao Hao

**Affiliations:** 1 Department of Medical Statistics, School of Public Health, Sun Yat-sen University, Guangzhou, 510080 Guangdong China; 2 Guangdong Mental Health Center, Guangdong Provincial People’s Hospital, Guangdong Academy of Medical Sciences, Guangzhou, 510080 Guangdong China; 3 Sun Yat-sen Global Health Institute, Sun Yat-sen University, Guangzhou, 510080 Guangdong China; 4 Center for Public Health and Epidemic Preparedness & Response, Peking University, Beijing, 100191 China

**Keywords:** Schizophrenia, Aggressiveness, Violent crime, Suicide attempt, Influencing factor, Count model

## Abstract

**Background:**

Schizophrenia patients have increased risks of adverse outcomes, including violent crime, aggressiveness, and suicide. However, studies of different adverse outcomes in schizophrenia patients are limited and the influencing factors for these outcomes need clarification by appropriate models. This study aimed to identify influencing factors of these adverse outcomes by examining and comparing different count regression models.

**Methods:**

This study included schizophrenia patients who had at least one follow-up record in the Guangdong Mental Health Center Network Medical System during 2020. Three types of adverse outcomes were included: a) aggressiveness with police dispatch or violent crime, b) aggressiveness without police dispatch, and c) self-harm or suicide attempts. The incidence density of these adverse outcomes was investigated using the Poisson, negative binomial (NB), zero-inflated Poisson (ZIP), and zero-inflated negative binomial (ZINB) models, accordingly. The best model was chosen based on goodness-of-fit tests. We further analyzed associations between the number of occurrences of adverse outcomes and sociodemographic, clinical factors with the best model.

**Results:**

A total of 130,474 schizophrenia patients were enrolled. Adverse outcomes rates were reported to be less than 1% for schizophrenia patients in 2020, in Guangdong. The NB model performed the best in terms of goodness-of-fit and interpretation when fitting for the number of occurrences of aggressiveness without police dispatch, whereas the ZINB models performed better for the other two outcomes. Age, sex, and history of adverse outcomes were influencing factors shared across these adverse outcomes. Higher education and employment were protective factors for aggressive and violent behaviors. Disease onset aged ≥ 18 years served as a significant risk factor for aggressiveness without police dispatch, and self-harm or suicide attempts. Family history of mental diseases was a risk factor for self-harm or suicide attempts individually.

**Conclusions:**

NB and ZINB models were selected for fitting the number of occurrences of adverse outcomes among schizophrenia patients in our studies. Influencing factors for the incidence density of adverse outcomes included both those shared across different types and those individual to specific types. Therefore, comprehensive and customized tools in risk assessment and intervention might be necessary.

## Background

Schizophrenia is a severe mental disorder characterized by delusions, hallucinations, impaired motivation, reduction in spontaneous speech, and social withdrawal [1]. According to the Global Burden of Disease Study 2019, the worldwide prevalence of schizophrenia was 0.32% whereas China’s prevalence was slightly higher, reaching 0.4% [2]. Besides prevalence burden, schizophrenia was associated with a weighted average of 14.5 years of potential life lost [3]. Schizophrenia patients are at an increased risk of several adverse outcomes, such as aggression, crime, suicide attempt, and suicide. The odds ratio of violent crime, suicide, and premature mortality for patients with schizophrenia and related disorders was over 7.5 when compared with the general population [4]. The prevalence of any aggression assessed by MOAS (Modified Overt Aggression Scale) in schizophrenia patients was above 30% [5, 6]. In terms of aggression that have more public health and public safety importance, the rate of physical violence was about 3% in China [7], whereas the rate of violent offence was 0.8% among women patients in Sweden [4]. The frequency of experiencing physical violence in their primary caregivers was 75.8% [8], while the lifetime risk of suicide attempts was 40–50% [9, 10]. Therefore, these adverse outcomes represent an essential public health issue and pose a great challenge to clinical treatment and patients’ health. Fortunately, these adverse events are considered to be preventable if intervention towards relevant influencing factors is carefully tailored. Thus, there remains a need and it is necessary to develop effective assessment methods for revealing influencing factors among schizophrenia patients with an inclination of adverse outcomes.

Many studies investigated the influencing factors for these adverse outcomes among schizophrenia patients. Being male and early in age were the common risk factors [11–13]. Other socio-demographic characteristics also serve as the risk factors for violence, including unemployment, lower levels of education, single status, and lower income [11, 14, 15]. Previous studies suggested that these adverse outcomes were also associated with a history of adverse outcomes, drug use, and a family history of psychiatric illness [13–15]. Other significant clinical factors including hospitalizations, lower satisfaction, and treatment adherence had been proposed in previous studies [12, 14, 16]. However, to the best of our knowledge, these studies only provided limited evidence concerning influencing factors for different types of adverse outcomes within the same population. Investigation of individual and shared influencing factors for the occurrences of adverse outcomes may contribute to risk assessment for these mental patients and shed a light on the prevention and treatment of schizophrenia.

In addition, patients may suffer from these events several times during the specified period, which means re-occurrence of outcomes within the same individual should be considered as count data. However, most studies treated these outcomes as dichotomous data. For instance, the MOAS score has been used as the dichotomous data for comparison between those reporting no aggressiveness (MOAS score equals 0) and those reporting aggressiveness (MOAS score greater than 0) without thinking about the re-occurrence of aggression behaviors. However, the re-occurrence of aggression behaviors indicates the level of aggressiveness, and previous studies suggested that higher aggressive levels were associated with severer psychiatric symptoms, thereby influencing the treatment and management of schizophrenia patients [15, 17]. Treating the variable as a simple dichotomous variable may result in loss of information and a decrease in statistical power. Therefore, treating the number of adverse outcomes as a count data, and identifying important factors of the incidence density of these events in patients with schizophrenia is necessary.

The Poisson regression model is commonly used to model count data, but its assumption of equal mean and variance is not reasonable in some cases. For overdispersed count data (greater variance compared with mean), the negative binomial regression is more appropriate [18, 19]. However, the above models cannot handle count data with excess zeros. The zero-inflated regression models considered the data as a mixture of a “zero” subset and another “non-zero” subset following specific distribution to address excess zeros [20, 21]. There are few studies investigating and empirically comparing the performance of different count models in terms of modeling adverse events among schizophrenia patients. In this study, we aimed to identify influencing factors that lead to different types of adverse outcomes, whether they are shared or individual related to these outcomes, by examining and comparing different count regression models.

## Methods

### Study participants

The study participants were enrolled from the Guangdong Mental Health Center Network Medical System (GDMHS), which was a disease registration report system based on the community mental health service network in Guangdong, China. GDMHS covered over 99% of schizophrenia patients in the province. There were over 348,000 patients registered in GDMHS during the periods from 2010 to 2019 in Guangdong, one of the biggest provinces in China [22]. Patient profile, clinical characteristics, and treatment information were recorded and maintained into GDMHS. Psychiatrist doctors and chief physicians were responsible for the data validation. To ensure data reliability, the Guangdong Health Commission conducted verification on the dataset annually by sampling surveys. The present study included schizophrenia patients with International Classification of Diseases, Tenth Revision code F20* in GDMHS. Other inclusion criteria were as follows: (1) the age of onset between 6–65 years of age; (2) having at least one follow-up during the period from January 1, 2020, to December 31, 2020; (3) no missing or implausible data. Patients with psychiatric comorbidities (including intellectual disabilities, alcohol or substance abuse, major depressive disorder, bipolar disorder) or other severe neurological diseases (including neurodegenerative diseases, cerebrovascular diseases, neurological tumors, neurological infectious diseases) were not included in the present study.

### Measures

We classified adverse outcomes according to different characteristics of behaviors, victim objects, interventions as well as services. The first outcome is the aggressiveness with police dispatch or violent crime, which is aggression or violence against others and leads to police dispatch or violation of Chinese law. The public security department was responsible for identifying aggressiveness with police dispatch and violent crime. The second outcome is aggressiveness without police dispatch, which is aggression or violence against others but does not lead to police dispatch. The third outcome is self-harm or suicide attempts. Aggressiveness without police dispatch, self-harm, and suicide attempts were reported by patients or their caregivers and assessed by community public health service workers. The total numbers of each outcome in 2020 were considered as the dependent variables.

Independence variables included sex, age, register type, education level, employment status, marital status, residential type, economic status, other medical history, family history of mental diseases, duration of illness, psychosis treatment status, duration of untreated psychosis, onset age, history of adverse outcomes. Information regarding independence variables was collected at the time of patient registration in GDMHS. All the categorical variables were entered into the models as the dummy variables.

### Analysis methods

The adverse outcome and independent variables were analyzed using descriptive analysis. Then, the Poisson regression, negative binomial (NB) regression, zero-inflated Poisson (ZIP) regression, and zero-inflated negative binomial (ZINB) regression were used to fit the data without covariates for each adverse outcome. The Likelihood Ratio test was used to identify overdispersion. The Vuong test was used for the non-nested model to determine whether there were excess zeros. In addition, the fitting goodness of each regression was evaluated by the difference between predicted and observed probabilities, log-likelihood, and Akaike information criterion (AIC). We selected the best fit model for multivariate regression for all adverse outcomes. We used backward stepwise selection as the variable selection method in our study. The logit part of ZIP and ZINB regression included intercept and the non-zero part included the same covariates as the Poisson and NB regression. Statistical significance was defined as *p* < 0.05. All analyses were conducted using R, version 4.0.

## Results

As shown in Table 1, a total number of 130,474 schizophrenia patients was enrolled in this study, and the adverse outcomes were reported by less than 1% of schizophrenia patients in 2020, in Guangdong, China. The majority of schizophrenia patients have not reported any adverse events in 2020. Only about 0.2%, 0.7%, and 0.1% of schizophrenia patients reported aggressiveness with police dispatch or violent crime, aggressiveness without police dispatch, and self-harm or suicide attempts, respectively.

**Table 1:** Number and proportion of adverse outcomes

Number of adverse outcomes	Aggressiveness with police dispatch or violent crime	Aggressiveness without police dispatch	Self-harm or suicide attempts
0	130,219 (99.805)	129,502 (99.255)	130,400 (99.943)
1	179 (0.137)	640 (0.491)	63 (0.048)
2	35 (0.027)	154 (0.118)	6 (0.005)
3	13 (0.010)	58 (0.044)	2 (0.002)
4	5 (0.004)	28 (0.021)	2 (0.002)
≥ 5	23 (0.018)	92 (0.071)	1 (0.001)
total	130,474(100)	130,474(100)	130,474(100)

Data are n (%)

Table 2 shows the distribution of independent variables. The mean age in schizophrenia patients was 47.6 ± 13.9 years with a range from 9.25 years to 99.8 years. Compared with all enrolled patients, those who had adverse events were early in age. Schizophrenia patients who reported self-harm or suicide attempts had the lowest age (35.9 ± 13.4 years). Among schizophrenia patients, 54.0% were males and larger proportions of males were found in all adverse outcomes. Most of the patients (87.7%) were registered. Among these patients, 85.5% had education levels of junior high school education or below. About 43.7% of the patients were unemployed. Aggressiveness with police dispatch or violent crime cases had a lower proportion of unemployment (40.4%), while aggressiveness without police dispatch cases (47.0%) and self-harm or suicide attempts cases (60.8%) had a higher proportion. Among schizophrenia patients, 51.3% of them were married, 64.3% of them lived in rural areas, and 41.3% of them had personal income lower than the local poverty level. Compared with total patients, those reporting aggressiveness with police dispatch or violent crime (74.5%), and aggressiveness without police dispatch cases (66.4%) were more likely to live in rural areas. Aggressiveness with police dispatch or violent crime cases had a lower proportion of living with poverty (29.0%) compared with overall patients. Only a small proportion of patients had a medical history (3.0%), and a family history of mental diseases (6.4%). Aggressiveness with police dispatch or violent crime cases had the lowest proportion of other medical history (0.4%), whereas self-harm or suicide attempts cases had the highest proportion of family history of mental diseases (17.6%). The majority of patients (82.9%) had their age at onset of schizophrenia at ≥ 18 years, but the cases of three outcomes had less proportion, with 79.6%, 80.3%, and 74.3% respectively. The mean duration of untreated psychosis was 4.17 ± 7.78 years (range 0–73 years), while the mean duration of illness was 19.2 ± 11.1 years (range 0.2–89.3 years). All types of adverse events cases had a shorter duration of illness as well as a shorter duration of untreated psychosis. Most of the patients received treatment (99.7%) and had adverse outcomes history (86.1%). However, patients reporting adverse outcomes had less proportion of adverse outcomes history, with 65.1%, 62.8%, and 60.8% respectively.

**Fig. 1:**
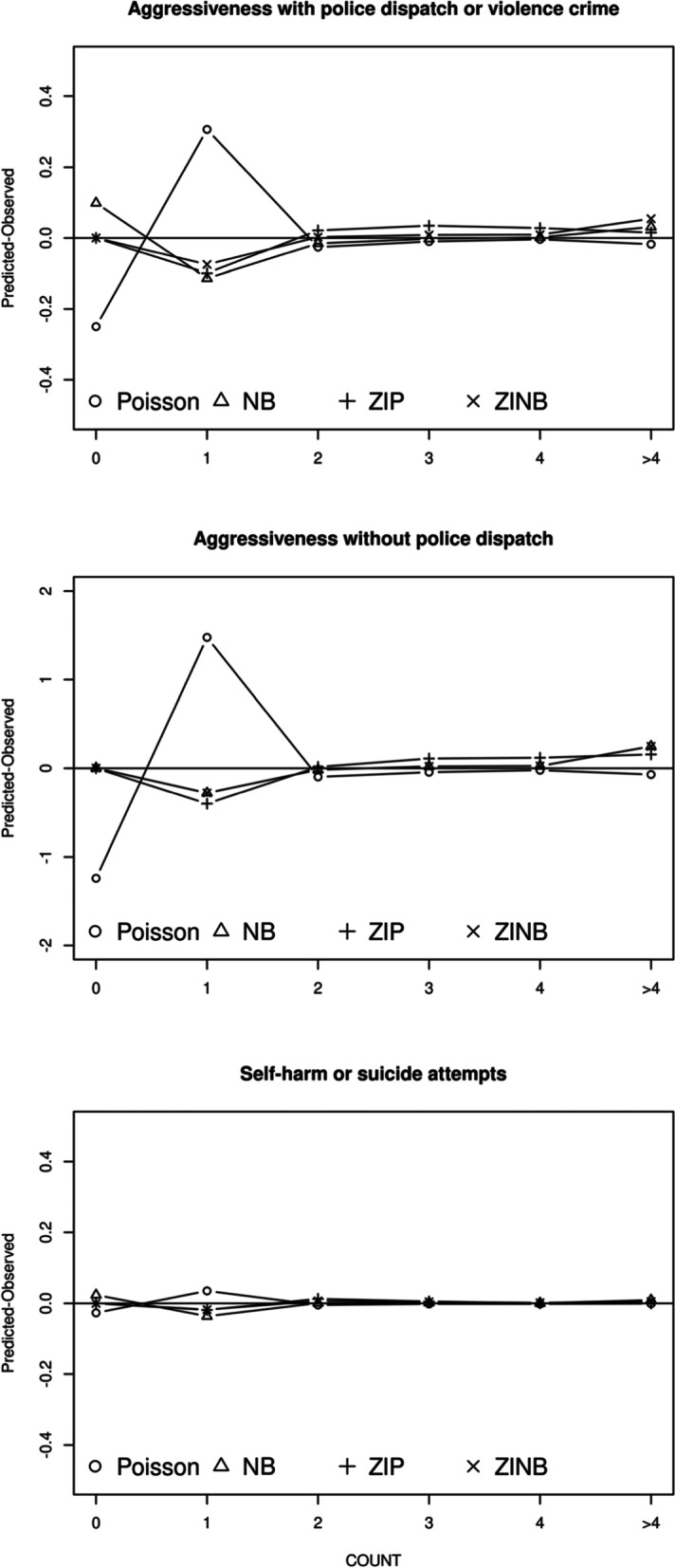
Predicted minus observed probabilities for four intercept-only models. NB, negative binomial; ZIP, zero-inflated Poisson; ZINB, zero-inflated negative binomial

Figure 1 shows the difference between the predicted and observed probabilities for each intercept-only model fitted to the number of adverse outcomes. It suggested that the Poisson model was a poor fit for all three outcomes. This model underestimated the probabilities of zeros and overestimated the probabilities of ones. The ZIP model performed better than the Poisson model, but the ZIP model predicted less 1 s and more > 1 s when fitting for the number of occurrences of aggressive and violent behaviors. The NB and ZINB models produced better fit when compared to the ZIP model, for the outcome of aggressiveness without police dispatch. The NB model overestimated the probabilities of zeros and underestimated the probabilities of ones in fitting the number of occurrences of aggressiveness with police dispatch or violent crime as well as in fitting the number of occurrences of self-harm or suicide attempts, while the ZINB model gave the best fit. The significant likelihood ratio test (*p* < 0.001, Table 3) showed that the number of counts of events was overdispersed. The Vuong test indicated that the ZIP model was favored over the Poisson (*p* < 0.001) when fitting for the number of occurrences of aggressive and violent behaviors. It also indicated that the ZINB model was favored over the NB model (*p* < 0.05) when fitting for the number of occurrences of aggressiveness with police dispatch or violent crime and for self-harm or suicide attempts. In addition, the value of Log-likelihood and AIC showed similar results. Taking the goodness-of-fit and model interpretation into account, the ZINB model was the best choice in modeling the number of occurrences of aggressiveness with police dispatch or violent crime, and the number of occurrences of self-harm or suicide attempts, while the NB model was the final model for fitting the number of occurrences of aggressiveness without police dispatch.

**Table 2:** Descriptive statistics for the characteristics of schizophrenia patients, grouped by each adverse outcome

Variable	Total Patients (*n* = 130,474)	Aggressiveness with police dispatch or violent crime cases (*n* = 255)	Aggressiveness without police dispatch cases (*n* = 972)	Self-harm or suicide attempts cases (*n* = 74)
Age(years)	47.6 ± 13.9	42.0 ± 12.5	42.9 ± 12.9	35.9 ± 13.4
Duration of untreated psychosis (years)	4.17 ± 7.78	2.84 ± 5.05	3.29 ± 6.34	2.54 ± 5.32
Duration of illness (years)	19.2 ± 11.1	15.7 ± 9.46	17.0 ± 10.1	13.6 ± 9.75
Sex				
Male	70,441 (54.0)	175 (68.6)	671 (69.0)	42 (56.8)
Female	60,033 (46.0)	80 (31.4)	301 (31.0)	32 (43.2)
Register type				
Register	114,390 (87.7)	228 (89.4)	857 (88.2)	58 (78.4)
Non-register	16,084 (12.3)	27 (10.6)	115 (11.8)	16 (21.6)
Educational level				
No education	19,933 (15.3)	35 (13.7)	92 (9.5)	6 (8.1)
Primary education	47,181 (36.2)	89 (34.9)	290 (29.8)	19 (25.7)
Junior high school education	44,329 (34.0)	93 (36.5)	408 (42.0)	31 (41.9)
High school education	11,180 (8.6)	26 (10.2)	102 (10.5)	8 (10.8)
Higher education	7851 (6.0)	12 (4.7)	80 (8.2)	10 (13.5)
Employment status				
Unemployment	57,007 (43.7)	103 (40.4)	457 (47.0)	45 (60.8)
Employment	73,467 (56.3)	152 (59.6)	515 (53.0)	29 (39.2)
Marital status				
Single	53,400 (40.9)	136 (53.3)	522 (53.7)	47 (63.5)
Married	66,990 (51.3)	93 (36.5)	364 (37.4)	23 (31.1)
Widowed	3786 (2.9)	2 (0.8)	21 (2.2)	1 (1.4)
Divorced	6298 (4.8)	24 (9.4)	65 (6.7)	3 (4.1)
Residential type				
Urban	46,555 (35.7)	65 (25.5)	327 (33.6)	28 (37.8)
Rural	83,919 (64.3)	190 (74.5)	645 (66.4)	46 (62.2)
Economic status				
Non-poverty	76,546 (58.7)	181 (71.0)	549 (56.5)	35 (47.3)
Poverty	53,928 (41.3)	74 (29.0)	423 (43.5)	39 (52.7)
Medical history				
Yes	3925 (3.0)	1 (0.4)	37(3.8)	2 (2.7)
No	126,549 (97.0)	254 (99.6)	935 (96.2)	72 (97.3)
Family history of mental diseases				
Yes	8533 (6.6)	20 (7.8)	94 (9.7)	13 (17.6)
No	120,697 (93.4)	235 (92.2)	878 (90.3)	61 (82.4)
Onset age(years)				
< 18 years	22,364 (17.1)	52 (20.4)	191 (19.7)	19 (25.7)
≥ 18 years	108,110 (82.9)	203 (79.6)	781 (80.3)	55 (74.3)
Psychosis treatment				
Yes	130,030 (99.7)	255 (100)	968 (99.6)	74 (100)
No	444 (0.3)	0 (0)	4 (0.4)	0 (0)
Adverse outcomes history				
Yes	112,329 (86.1)	166 (65.1)	610 (62.8)	45 (60.8)
No	18,145 (13.9)	89 (34.9)	362 (37.2)	29 (39.2)

Data are mean ± SD for continuous variables

n(%) for categorical variables

**Table 3:** The results of goodness-of-fit statistics and tests for four intercept-only models

Models	Log likelihood	AIC	The likelihood ratio test (*p*-Value)^a^	The Vuong test (*p*-Value)^b^
Aggressiveness with police dispatch or violent crime				
Poisson	-4827.2	9656.4	4893.7**(< 0.001)**	-4.2**(< 0.001)**
NB	-2380.3	4764.7	—	-4.1**(< 0.001)**
ZIP	-2870.0	5744.1	1121.8**(< 0.001)**	—
ZINB	-2309.1	4624.3	—	—
Aggressiveness without police dispatch				
Poisson	-18,726.1	37,454.2	22,152.0**(< 0.001)**	-8.6**(< 0.001)**
NB	-7650.2	15,304.4	—	**-**0.002 (= 0.499)
ZIP	-10,786.3	21,576.6	6272.2**(< 0.001)**	—
ZINB	-7650.2	15,306.4	—	—
Self-harm or suicide attempts				
Poisson	-990.9	1983.7	513.4**(< 0.001)**	-1.6(= 0.054)
NB	-734.2	1472.4	—	-1.7**(= 0.046)**
ZIP	-762.7	1529.3	92.8**(< 0.001)**	—
ZINB	-716.3	1438.6	—	—

NB negative binomial, ZIP zero-inflated Poisson, ZINB zero-inflated negative binomial

^a^the likelihood ratio test for overdispersion (Poisson vs. NB and ZIP vs. ZINB)

^b^the Vuong test for excess zeros (Poisson vs. ZIP and NB vs. ZINB)

**Fig. 2:**
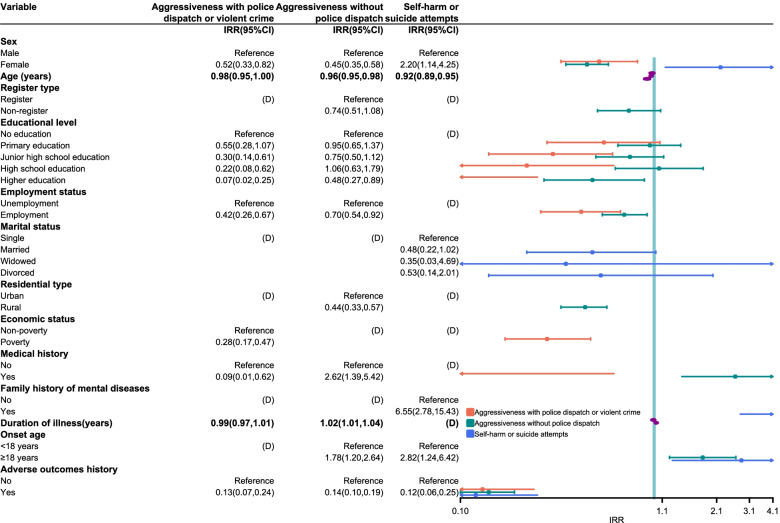
Multivariate count regression models for the number of occurrences of adverse outcomes among schizophrenia patients. Zero-inflated Negative Binomial regression was used to model the number of occurrences of aggressiveness with police dispatch or violent crime, and to the number of occurrences of self-harm or suicide attempts, count model regression results were displayed. Negative Binomial regression was used to model the number of occurrences of aggressiveness without police dispatch. (D), Excluding from model

The results of multivariate count regression models are shown in Fig. 2. Sex, age, and adverse outcomes history were associated with all types of adverse outcomes. The number of occurrences of aggressiveness with police dispatch or violent crime and the number of occurrences of aggressiveness without police dispatch for female patients was lower than that for males patients, with incidence rate ratios (IRR) being 0.52 (95% CI: 0.33,0.82; *p* = 0.005) and 0.45 (95% CI: 0.35,0.58; *p* < 0.001) respectively. On the other hand, being female was a risk factor for self-harm or suicide attempts (IRR = 2.20; 95% CI: 1.14,4.25; *p* = 0.019). Elderly patients and patients having adverse outcomes history showed less risk in three adverse outcomes. Patients who had higher educational levels exhibited a lower likelihood of aggressiveness with police dispatch or violent crime. Having higher education (IRR = 0.48; 95% CI: 0.27, 0.89; *p* = 0.010) also decreased the number of occurrences of aggressiveness without police dispatch compared to those who had received no education. There was an association between unemployment and an increased number of occurrences of aggressiveness with police dispatch or violent crime, and that of occurrences of aggressiveness without police dispatch. Living in rural (IRR = 0.44; 95% CI: 0.33, 0.57; *p* < 0.001) and poverty (IRR = 0.28; 95% CI: 0.17, 0.49; *p* < 0.001) was associated with reduced risk of aggressiveness without police dispatch, and aggressiveness with police dispatch or violent crime respectively. Having medical history was significantly associated with a higher risk of aggressiveness without police dispatch (IRR = 2.62; 95% CI: 1.39,5.42; *p* = 0.002), and significantly associated with lower risk of aggressiveness with police dispatch or violent crime (IRR = 0.28; 95% CI: 0.17,0.47; *p* < 0.001). Longer duration of illness (IRR = 1.02; 95% CI: 1.01,1.04; *p* = 0.002) and adult-onset schizophrenia (onset age ≥ 18 years) (IRR = 1.78; 95% CI: 1.20,2.64; *p* = 0.001) significantly increased the risk of aggressiveness without police dispatch. Adult-onset schizophrenia (IRR = 2.82; 95% CI: 1.24,6.42; *p* = 0.002) and having family history of mental diseases (IRR = 6.55; 95% CI: 2.78,15.43; *p* < 0.001) were risk factors for self-harm or suicide attempts.

## Discussion

To the best of current knowledge, though count models have been implemented in many epidemiological and public health studies, our study is the first of its kind to utilize count regression models to analyze the incidence density of the adverse outcomes in schizophrenia patients [23–26]. Moreover, it is crucially important to select the best-fitted model for the data since there is no model fitted well for all data. Here, we used four count regression models to fit the incidence density of the adverse outcomes in schizophrenia patients and investigated influencing factors associated with the number of occurrences of adverse outcomes by the best-fitted model. In our study, about 1% of schizophrenia patients have reported adverse outcomes in 2020. Traditional Poisson regression had the worst fit to this study data for all types of outcomes because of the overdispersion and excess zeros in the present study. To solve the zero-inflated problem, the ZIP model was used which was an obvious choice over Poisson regression. To deal with the overdispersion problem, the NB model was used and also fitted the data better than the Poisson model. However, the NB model can not yet solve the zero-inflated issue. Hence, the ZINB model was used, which took both overdispersion and the excess zeroes into account. The ZINB model can also provide a similar fit compared to the NB model because no zero-inflated issue was depicted when using the NB model for aggressiveness without police dispatch. Therefore, the NB model was selected to be the simplest and first choice to fit the number of aggressiveness without police dispatch because of no zero-inflated issue, whereas the ZINB model was preferred for the other two outcomes to account for both overdispersion and the excess zeroes.

Investigation of influencing factors for adverse outcomes is of benefit to intervention and prevention of schizophrenia. Specifically, epidemiological knowledge may translate into clinical practice by developing clinically feasible assessment tools to predict risk and support the clinical judgement. Interventions, as well as preventions, can be targeted at those likely to have a risk of adverse outcomes. In our study, a wide range of sociodemographic and clinical factors were assessed. Our finding indicated that being early in age increased the risk of all outcomes, including aggressiveness with police dispatch or violent crime, aggressiveness without police dispatch, and self-harm or suicide attempts. These results are consistent with the results of previous studies that have explored risk factors with binomial response [11, 12, 27, 28]. The risk of adverse events for violence against others is increased for males, being consistent with those conducted by Fazel et al. [29]. When self-harm or suicide attempts are strictly defined as physical violence against oneself, the risk of self-harm or suicide attempts is increased for females, which is also consistent with previous studies [30, 31].

In addition, our findings revealed that having history of adverse outcomes significantly decreases the likelihood of all three outcomes. It is contrary to the finding of previous studies [15, 30, 32, 33]. It is worth noting that the schizophrenia patients in our study were all provided with primary public health care freely offered by the Chinese government [34]. They received regular follow-up and intervention by community mental health professionals. Patients who had the previous report of adverse outcomes had higher scores of risk assessments, and thus received more frequent follow-up services [22]. Moreover, patients’ information about adverse outcomes can be shared with public security departments, by which the occurrences of adverse outcomes can be prevented from having a negative impact on the social harmony and stability. Therefore, not only their caregivers, but also public security personnel may take on a more active role in helping schizophrenia patients with behavioral issues to prevent the recurrence of adverse outcomes. Besides, their psychiatrists may adjust drug doses or modify treatment regimens according to the mental health condition, thereby leading to remission. Therefore, having a history of adverse outcomes may decrease the risk of adverse events because of the above intervention. However, our inference for the relationship between the above-mentioned intervention and each adverse outcome may still need further exploration in the future study.

However, results from our study showed that many risk factors were not shared across the adverse outcomes. It suggests that it is distinct for mechanism between violence and self-harm or suicide attempts, and customized tools in risk assessment and intervention for specific events are necessary. It also provides a priori knowledge for risk assessment in clinical practice. For example, educational level, employment status, and medical history were related to violence against others but not to self-harm and suicide attempts. Our findings were in concordance with the results of previous studies which reported unemployment status and low educational level as risk factors for violence [17, 35, 36]. Employment status and educational level should be one of the most important factors considered by clinicians when assessing the risk of violence. Whiting et al. reported that having medical history increases the risk of violence [37]. However, our study found a negative relationship between medical history and aggressiveness with police dispatch or violent crime. The possible reasons may be similar to the association between history of adverse outcomes and violence. Having medical history may increase the risk of adverse effects [38–40]. When patients had an adverse effect, he or she may be evaluated as having an unstable mental condition and thus received more intensive follow-up services and symptomatic treatment, thereby contributing to the decreases of their occurrence of violence.

Having a family history of mental disease increased the risk of self-harm or suicide attempts but did not affect violent behaviors. Several previous research found that family history of mental disease was a significant risk factor for self-harm or suicide attempts [13, 41–43], but suggested no conclusive finding in terms of violence [44]. Our findings, consistent with many others, support that self-harm or suicide attempts may be affected by genetic factors [45, 46]. Although previous studies found that earlier age of onset was a risk factor of violence and suicide, we found that adult-onset schizophrenia increases the risk of aggressiveness without police dispatch and the risk of self-harm or suicide attempts. This is in line with previous studies, suggesting that adult-onset patients are more susceptible to schizophrenia and may have more traits for passive as well as aggressive personality disorder compared with early-onset patients [30, 47, 48]. Further studies are warranted to address the relationship between the density of events and the age of illness onset.

In our study, patients who had a longer duration of illness were more likely to conduct aggressiveness without police dispatch, but were unlikely to lead to other outcomes. However, schizophrenia patients with a history of severe violence or with suicide attempts had a longer duration of illness in other studies [31, 49, 50]. It is possible that, for patients with a longer duration of illness, the caregivers, as well as community mental health professionals, may have a better awareness of their much more serious outcomes (for example, suicide attempts and violent crime). Based on these notions, we argue that the caregivers and the other relevant stakeholders may be in a more active role in preventing occurrences of more serious outcomes. Further studies are needed to clarify the relationship between the duration of illness and different types of adverse outcomes.

Additionally, the residential type was another individual influencing factor for aggressiveness without police dispatch, in which living in rural was found to be a protective factor. A possible explanation is that rural schizophrenia patients are much more inclined to receive primary public health care and have better access to primary medical institutions in China, which may contribute to achieving effective control and decreasing the risk of violence [51, 52]. The different clinical strategies of treatment of schizophrenia between urban and rural areas may be another possible explanation. A previous study in China found that rural patients were more likely to receive anticholinergics compared to urban patients [53]. Similarly, poverty was an individual protective factor for aggressiveness with police dispatch or violent crime. This may be attributed to the higher rate of receiving regular follow-ups and increasing demand and utilization of health services among patients under the poverty lines [51, 54].

In our study, some factors were not significantly associated with the number of occurrences of adverse outcomes. For instance, psychosis treatment status showed an insignificant association with the number of occurrences of these outcomes. The fact that there were too few untreated patients in our study resulted in insufficient statistical power to detect the differences. Other factors, such as marital status, register type, were not related to the number of occurrences of adverse outcomes. It may be attributed to the different statistical analysis methods. In the count regression model, the exponentiated regression coefficient of the count model is the ratio of expected counts instead of the odds ratio in the logistic regression model. In other words, the exponentiated coefficient represents the IRR for each unit change in the predictor, while the other predictors in the model are held constant. Another possible reason for the non-significance is the differences in the definition of these adverse outcomes and the research population. Further studies should be needed to assess the impact of varying covariates on the incidence density of these events among schizophrenia patients.

Several limitations should be noted. First, the inclusion and exclusion criteria of patients may introduce selection bias, but the demographics distribution of enrolled patients in our study was similar to that of the schizophrenia patients followed up in 2020. Secondly, moderate correlation between some given independent variable would generally not result in severe multicollinearity, and thus there is no need for additional attention for such groups of covariates in terms of our regression problem. Finally, our results were limited due to the definition of adverse outcomes and the lack of variables that may contribute to adverse outcomes (for example, scales, antipsychotic drugs, drug adherence, parental abuse, and clinical symptoms).

## Conclusions

Count regression model not only assesses the presence or absence of adverse outcomes but also the incidence density of it. We compared the model performance of the Poisson, NB, ZIP, and ZINB regression models to investigate the distributed nature of three types of adverse outcomes in schizophrenia patients and selected the NB and ZINB as the best models. Several shared influencing factors across different adverse outcomes were detected, such as age, sex, and history of adverse events. However, many factors were specific only to some adverse outcomes, which suggests that risk assessment and intervention might have to be tailored for specific outcomes. Moreover, some influencing factors were inconsistent with previous studies because of the different analysis models, study population, definition and measurement of adverse outcome, and intervention of community public health. It is necessary to translate epidemiological knowledge into comprehensive and customized tools to assess and manage the risk of adverse outcomes, thereby improving medical care and public safety.

## Data Availability

The data that support the findings of this study are available from Guangdong Mental Health Center but restrictions apply to the availability of these data, which were used under license for the current study, and so are not publicly available. Data are however available from the authors upon reasonable request and with permission of Guangdong Mental Health Center.

## Abbreviations

GDMHS: Guangdong Mental Health Center Network Medical System
NB: Negative binomial
ZIP: Zero-inflated Poisson
ZINB: Zero-inflated negative binomial
AIC: Akaike information criterion

## References

[1] Owen MJ, Sawa A, Mortensen PB (2016). Schizophrenia. Lancet.

[2] Global Health Data Exchange. GBD Results Tool. Available from: http://ghdx.healthdata.org/gbd-results-tool. Accessed 13 May 2021.

[3] Hjorthøj C, Stürup AE, McGrath JJ, Nordentoft M (2017). Years of potential life lost and life expectancy in schizophrenia: a systematic review and meta-analysis. Lancet Psychiatry.

[4] Fazel S, Wolf A, Palm C, Lichtenstein P (2014). Violent crime, suicide, and premature mortality in patients with schizophrenia and related disorders: a 38-year total population study in Sweden. Lancet Psychiatry.

[5] Wu YQ, Kang RY, Yan YX, Gao KM, Li ZW, Jiang J, Chi XY, Xia LL (2018). Epidemiology of schizophrenia and risk factors of schizophrenia-associated aggression from 2011 to 2015. J Int Med Res.

[6] Li W, Yang Y, Hong L, An FR, Ungvari GS, Ng CH, Xiang YT (2020). Prevalence of aggression in patients with schizophrenia: a systematic review and meta-analysis of observational studies. Asian J Psychiatr.

[7] Sun L, Han X, Wang K, Xu C, Song Z, Zhang J, Cao D, Tan L, Chen F, Wu S, He L, Wan C (2021). Candidate symptomatic markers for predicting violence in schizophrenia: a cross-sectional study of 7711 patients in a Chinese population. Asian J Psychiatr.

[8] Kageyama M, Solomon P, Yokoyama K, Nakamura Y, Kobayashi S, Fujii C (2018). Violence towards family caregivers by their relative with schizophrenia in Japan. Psychiatr Q.

[9] Duko B, Ayano G (2018). Suicidal ideation and attempts among people with severe mental disorder, Addis Ababa, Ethiopia, comparative cross-sectional study. Ann Gen Psychiatry.

[10] Beck-Felts K, Goodman M, Ospina LH, Wall M, McEvoy J, Jarskog LF, Ballon JS, Bartels MN, Buchsbaum R, Sloan RP (2020). Suicide reduction in schizophrenia via exercise (SUnRISE): study protocol for a multi-site, single-blind, randomized clinical trial of aerobic exercise for suicide risk reduction in individuals with schizophrenia. Trials.

[11] Elbogen EB, Johnson SC (2009). The intricate link between violence and mental disorder. Arch Gen Psychiatry.

[12] Large MM, Nielssen O (2011). Violence in first-episode psychosis: a systematic review and meta-analysis. Schizophr Res.

[13] Cassidy RM, Yang F, Kapczinski F, Passos IC (2018). Risk factors for suicidality in patients with schizophrenia: a systematic review, meta-analysis, and meta-regression of 96 studies. Schizophr Bull.

[14] Mauri MC, Cirnigliaro G, Di Pace C, Paletta S, Reggiori A, Altamura CA, Dell'Osso B (2019). Aggressiveness and violence in psychiatric patients: a clinical or social paradigm?. CNS Spectr.

[15] Lopez-Garcia P, Ashby S, Patel P, Pierce KM, Meyer M, Rosenthal A, Titone M, Carter C, Niendam T (2019). Clinical and neurodevelopmental correlates of aggression in early psychosis. Schizophr Res.

[16] Bobes J, Fillat O, Arango C (2009). Violence among schizophrenia out-patients compliant with medication: prevalence and associated factors. Acta Psychiatr Scand.

[17] Caqueo-Urizar A, Fond G, Urzua A, Boyer L, Williams DR (2016). Violent behavior and aggression in schizophrenia: prevalence and risk factors. A multicentric study from three Latin-America countries. Schizophr Res.

[18] Allison PD. Logistic Regression Using the SAS System: Theory and Application. Wiley-SAS, Cary NC USA; 2001.

[19] Byers AL, Allore H, Gill TM, Peduzzi PN (2003). Application of negative binomial modeling for discrete outcomes. J Clin Epidemiol.

[20] Lambert D (1992). Zero-inflated poisson regression with an application to defects in manufacturing. Technometrics.

[21] William HG. Accounting for Excess Zeros and Sample Selection in Poisson and Negative Binomial Regression Models. NYU Working Paper. 1994:No. EC-94–10. https://ssrn.com/abstract=1293115.

[22] Tan W, Lin H, Lei B, Ou A, He Z, Yang N, Jia F, Weng H, Hao T (2020). The psychosis analysis in real-world on a cohort of large-scale patients with schizophrenia. BMC Med Inform Decis Mak.

[23] Zaninotto P, Falaschetti E (2011). Comparison of methods for modelling a count outcome with excess zeros: application to activities of daily living (ADL-s). J Epidemiol Community Health.

[24] Akbarzadeh Baghban A, Pourhoseingholi A, Zayeri F, Jafari AA, Alavian SM (2013). Application of zero-inflated poisson mixed models in prognostic factors of hepatitis C. Biomed Res Int.

[25] Sharareh P, Leili T, Abbas M, Jalal P, Ali G (2020). Determining correlates of the average number of cigarette smoking among college students using count regression models. Sci Rep.

[26] Kang KI, Kang K, Kim C (2021). Risk factors influencing cyberbullying perpetration among middle school students in Korea: analysis using the zero-inflated negative binomial regression model. Int J Environ Res Public Health.

[27] Dai Q, Wang D, Wang J, Xu H, Andriescue EC, Wu HE, Xiu M, Chen D, Zhang X (2020). Suicide attempts in Chinese Han patients with schizophrenia: cognitive, demographic, and clinical variables. Braz J Psychiatry.

[28] Olfson M, Stroup TS, Huang C, Wall MM, Crystal S, Gerhard T (2021). Suicide risk in medicare patients with schizophrenia across the life span. JAMA Psychiat.

[29] Fazel S, Wolf A, Larsson H, Lichtenstein P, Mallett S, Fanshawe TR (2017). Identification of low risk of violent crime in severe mental illness with a clinical prediction tool (Oxford mental illness and violence tool [OxMIV]): a derivation and validation study. Lancet Psychiatry.

[30] Reutfors J, Brandt L, Jonsson EG, Ekbom A, Sparen P, Osby U (2009). Risk factors for suicide in schizophrenia: findings from a Swedish population-based case-control study. Schizophr Res.

[31] Mork E, Walby FA, Harkavy-Friedman JM, Barrett EA, Steen NE, Lorentzen S, Andreassen OA, Melle I, Mehlum L (2013). Clinical characteristics in schizophrenia patients with or without suicide attempts and non-suicidal self-harm - a cross-sectional study. BMC Psychiatry.

[32] Ahmed AO, Richardson J, Buckner A, Romanoff S, Feder M, Oragunye N, Ilnicki A, Bhat I, Hoptman MJ, Lindenmayer JP (2018). Do cognitive deficits predict negative emotionality and aggression in schizophrenia?. Psychiatry Res.

[33] Fernandez-Sevillano J, Gonzalez-Pinto A, Rodriguez-Revuelta J, Alberich S, Gonzalez-Blanco L, Zorrilla I, Velasco A, Lopez MP, Abad I, Saiz PA (2021). Suicidal behaviour and cognition: a systematic review with special focus on prefrontal deficits. J Affect Disord.

[34] The National Health Commission of the People's Republic of China. Practices of Treatment and Management of Severe Mental illness. 2018. http://www.gov.cn/gongbao/content/2018/content_5338247.htm Accessed 12 Mar 2022.

[35] Karabekiroglu A, Pazvantoglu O, Karabekiroglu K, Boke O, Korkmaz IZ (2016). Associations with violent and homicidal behaviour among men with schizophrenia. Nord J Psychiatry.

[36] Hachtel H, Harries C, Luebbers S, Ogloff JR (2018). Violent offending in schizophrenia spectrum disorders preceding and following diagnosis. Aust N Z J Psychiatry.

[37] Whiting D, Gulati G, Geddes JR, Fazel S (2022). Association of schizophrenia spectrum disorders and violence perpetration in adults and adolescents from 15 Countries: a systematic review and meta-analysis. JAMA Psychiatry.

[38] De Hert M, Detraux J, van Winkel R, Yu W, Correll CU (2011). Metabolic and cardiovascular adverse effects associated with antipsychotic drugs. Nat Rev Endocrinol.

[39] Boden R, Edman G, Reutfors J, Ostenson CG, Osby U (2013). A comparison of cardiovascular risk factors for ten antipsychotic drugs in clinical practice. Neuropsychiatr Dis Treat.

[40] Nielsen RE, Banner J, Jensen SE (2021). Cardiovascular disease in patients with severe mental illness. Nat Rev Cardiol.

[41] Taylor PJ, Hutton P, Wood L (2015). Are people at risk of psychosis also at risk of suicide and self-harm? A systematic review and meta-analysis. Psychol Med.

[42] Capra C, Kavanagh DJ, Hides L, Scott JG (2015). Subtypes of psychotic-like experiences are differentially associated with suicidal ideation, plans and attempts in young adults. Psychiatry Res.

[43] DeVille DC, Whalen D, Breslin FJ, Morris AS, Khalsa SS, Paulus MP, Barch DM (2020). Prevalence and family-related factors associated with suicidal ideation, suicide attempts, and self-injury in children aged 9 to 10 years. JAMA Netw Open.

[44] Wang J, Zhang SM, Zhong SL, Mellsop G, Guo HJ, Li QG, Zhou JS, Wang XP (2019). Gender differences among homicide offenders with schizophrenia in Hunan Province. China Psychiatry Res.

[45] Maciejewski DF, Creemers HE, Lynskey MT, Madden PA, Heath AC, Statham DJ, Martin NG, Verweij KJ (2014). Overlapping genetic and environmental influences on nonsuicidal self-injury and suicidal ideation: different outcomes, same etiology?. JAMA Psychiat.

[46] Turecki G, Brent DA, Gunnell D, O'Connor RC, Oquendo MA, Pirkis J, Stanley BH (2019). Suicide and suicide risk. Nat Rev Dis Primers.

[47] Kim SW, Kim SJ, Mun JW, Bae KY, Kim JM, Kim SY, Yang SJ, Shin IS, Yoon JS (2010). Psychosocial factors contributing to suicidal ideation in hospitalized schizophrenia patients in Korea. Psychiatry Investig.

[48] Skokou M, Gourzis P (2014). Demographic features and premorbid personality disorder traits in relation to age of onset and sex in paranoid schizophrenia. Psychiatry Res.

[49] Chang QS, Wu DH, Rong H, Wu ZW, Tao WQ, Liu HM, Zhou P, Luo GZ, Xie GH, Huang SW (2019). Suicide ideation, suicide attempts, their sociodemographic and clinical associates among the elderly Chinese patients with schizophrenia spectrum disorders. J Affect Disord.

[50] Tesli N, van der Meer D, Rokicki J, Storvestre G, Rosaeg C, Jensen A, Hjell G, Bell C, Fischer-Vieler T, Tesli M (2020). Hippocampal subfield and amygdala nuclei volumes in schizophrenia patients with a history of violence. Eur Arch Psychiatry Clin Neurosci.

[51] Tian M, Wang H, Tong X, Zhu K, Zhang X, Chen X (2015). Essential public health services’ accessibility and its determinants among adults with chronic diseases in China. PLoS One.

[52] Wang X, Yang H, Duan Z, Pan J (2018). Spatial accessibility of primary health care in China: a case study in Sichuan Province. Soc Sci Med..

[53] Hou CL, Wang SB, Wang F, Xu MZ, Chen MY, Cai MY, Xiao YN, Jia FJ (2019). Psychotropic medication treatment patterns in community-dwelling schizophrenia in China: comparisons between rural and urban areas. BMC Psychiatry.

[54] Xu J, Zheng J, Xu L, Wu H (2021). Equity of Health Services Utilisation and Expenditure among Urban and Rural Residents under Universal Health Coverage. Int J Environ Res Public Health.

